# Digital quantification of nuclei in porcine experimental and forensic wounds: effects of wound age and body location on nuclei density

**DOI:** 10.1186/s13028-026-00866-5

**Published:** 2026-04-29

**Authors:** Kristiane Barington, Anders Læsø Madsen, Henrik Elvang Jensen

**Affiliations:** 1 Department of Veterinary and Animal Sciences, Faculty of Health and Medical Sciences, University of Copenhagen, Ridebanevej 3, Frederiksberg C, DK-1870 Denmark; 2 Department of Computer Science, The Technical Faculty of IT and Design, Aalborg University, Selma Lagerløfs Vej 300, DK9220, Aalborg, Denmark

**Keywords:** Age assessment, Cell detection, Porcine, Veterinary forensic pathology, Wound

## Abstract

**Background:**

Age assessment of wounds is a challenge in both human and veterinary forensic pathology. This study aimed to evaluate the density, dimensions, and circularity of nuclei in experimental and forensic porcine wounds over time by application of software for nucleus detection and characterization. Moreover, to evaluate whether the nuclei density is affected by the location of the wound on the body.

**Methods:**

HE-stained tissue sections of granulation tissue from 181 porcine wounds (*n* = 47 pigs) from a porcine excisional wound model were included, together with 30 porcine skin wounds (*n* = 22 pigs) received for forensic examination. In addition, tissue sections of intact skin from 13 experimental pigs were included as controls. The experimental wounds had healed by second intention and were divided into seven groups based on wound age (5, 10, 15, 20, 25, 30, and 35 days). Forensic wounds were divided into three age groups based on the age assessment stated in the forensic reports (days, > 1 week, or several weeks). Moreover, the forensic wounds, several weeks old, were divided into three groups based on body location (umbilical outpouching, limbs, and ear/tail). All tissue sections were digitized, and nuclei were quantified and measured (area, diameter, and circularity) by application of the InstanSeg extension in QuPath.

**Results:**

The density of nuclei in granulation tissue from the experimental wounds showed a significant decrease over time (*P* < 0.05, η^2^_P_ ≥ 0.88). In granulation tissue from forensic wounds, the nuclei density also showed a tendency of a time-dependent pattern, but this was not statistically significant (*P* > 0.05). Nuclei density was significantly lower in wounds located on umbilical outpouchings compared to wounds located on ears/tails (*P* < 0.05). Nucleus area, diameter, and circularity did show significant differences between age groups (*P* < 0.05, η^2^_H_ ≤ 0.03). However, effect sizes were too low and differences too small to be suitable for age assessment of wounds.

**Conclusion:**

Digital quantification of nuclei density is promising as a supportive tool for assessing wound age in veterinary forensic pathology. However, nuclei density can be affected by the body location of the wound and should not be used as a standalone method for age assessment.

**Supplementary Information:**

The online version contains supplementary material available at 10.1186/s13028-026-00866-5.

## Background

Veterinary pathologists are frequently asked by the police or other authorities to assess skin wounds as part of forensic examinations [1–3]. Especially, assessing the age of wounds is often crucial, as it may be used in a judicial proceeding to determine how long an animal has suffered or to assign responsibility, i.e., to establish which party had the custody of the animal when the wound was inflicted [2].

In Denmark, the majority of veterinary cases submitted for forensic examination involve pigs, and from 2020 to 2022, one-third of porcine cases concerned wounds as the primary lesion [2, 4]. Many studies of porcine experimental wounds have been published, but the majority focus on wound treatments, diabetic wounds, burns, and hypertrophic scarring and are, therefore, not comparable to forensic cases [5–7]. However, a porcine wound model was developed in 2018 and refined in 2026 to characterize granulation tissue over time [8, 9].

Currently, age assessment of wounds is based on gross and histological evaluation of granulation tissue [2, 10]. In pigs, granulation tissue is grossly visible in 5-day-old wounds, and its thickness increases 0.5 to 1.2 mm/day [8, 11]. However, experimental studies have shown that the thickness of granulation tissue may decrease after approximately 10 days [8, 9]. Therefore, thickness alone is not a reliable indicator of wound age. Typically, tissue sections of granulation tissue are stained with hematoxylin and eosin (HE). However, assessment of granulation tissue maturity based on HE-stained slides is subject to substantial interobserver variability and less intraobserver variability [9]. Histologically, newly formed granulation tissue is characterized by high cell density, and proliferating cells are often large and plump [9]. As granulation tissue is remodeled into fibrous scar tissue, cell density decreases, and fibroblasts become more elongated [9, 12]. Based on this, we hypothesize that measurements of nuclei density and shape could reflect wound age.

With recent advancements in software for evaluating tissue slides, digital pathology may be used for objective age assessment of wounds based on HE-stained tissue slides. QuPath is an open-source software for whole slide image analysis that offers the possibility for quantitative evaluation of tissue components [13]. Recently, a novel segmentation algorithm entitled InstanSeg was developed for accurate and efficient nuclei segmentation [14]. InstanSeg is open-source software and available as a user-friendly extension to QuPath that enables nuclei detection in brightfield or fluorescence images and measures the size and shape of the nuclei [14]. InstanSeg has recently been used for nucleus detection in lymphomas in humans [15]. In the present study, the hypothesis was that objective quantification of nuclei density, maximum diameter, and circularity in granulation tissue could be used for age assessment of skin wounds.

This study aimed to evaluate the density, area, diameter, and circularity of nuclei in granulation tissue from porcine experimental and forensic wounds over time by application of the InstanSeg extension in QuPath. Moreover, to evaluate whether the nuclei density is affected by the location of the wound on the body.

## Methods

### Experimental porcine wounds

HE-stained tissue sections of granulation tissue from 188 porcine wounds (*n* = 47 pigs) from a recent porcine excisional wound model were used [9]. In addition, tissue sections of intact skin from 13 experimental pigs were included as controls [9]. In summary, each pig (body weight: 20–25 kg) was anesthetized, and four full-thickness wounds measuring 2 × 2 cm were excised on the back. Wounds were left untreated to heal by second intention, and pigs were euthanized after 5, 10, 15, 20, 25, 30, and 35 days, with six or seven pigs in each group. Samples of wounds and intact skin were formalin-fixed, processed through stepwise concentrations of ethanol and xylene, and paraffin-embedded [9]. The experimental procedure was approved by the Danish Animal Inspectorate (2023-15-0201-01363).

### Porcine wounds from forensic cases

HE-stained tissue sections from 30 porcine skin wounds (*n* = 22 pigs) received for forensic examination at the University of Copenhagen in 2021 were evaluated. The cases were reported by veterinary enforcement officers undertaking meat inspection at slaughterhouses or carrying out inspections on farms. For 28 out of 30 wounds, the tissue had been scalded, singed, and scraped as part of the slaughter process. All cases had been frozen prior to the forensic examination. From the case reports, information regarding wound age, body location, thickness of granulation tissue, and fibrous tissue was extracted. Wound age was in all cases assessed by a single veterinary pathologist with more than 30 years of experience. Based on the wound age stated in the case report, wounds were allocated into three groups (1: days, 2: more than a week (> 1 week), or 3: several weeks). Moreover, the forensic wounds, several weeks old, were divided into three groups based on body location (A: umbilical outpouching, B: limbs, and C: ear/tail).

### Detection of nuclei

All tissue sections were digitized using an Axioscan 7 scanner with a 20x/0.8 objective (Zeiss, Germany) and imported into QuPath v0.6.0-rc3 setting the image type to Brightfield HE. Color deconvolution, i.e., separation of the stains (hematoxylin, eosin, and residual) was performed on each tissue slide. First, a 60 × 60 μm annotation was created in a white area to set the background vectors. Then, color deconvolution was performed by creating a 60 × 60 μm annotation in an area representing eosinophilic and basophilic stain and running the Estimate stain vectors command.

**Fig. 1:**
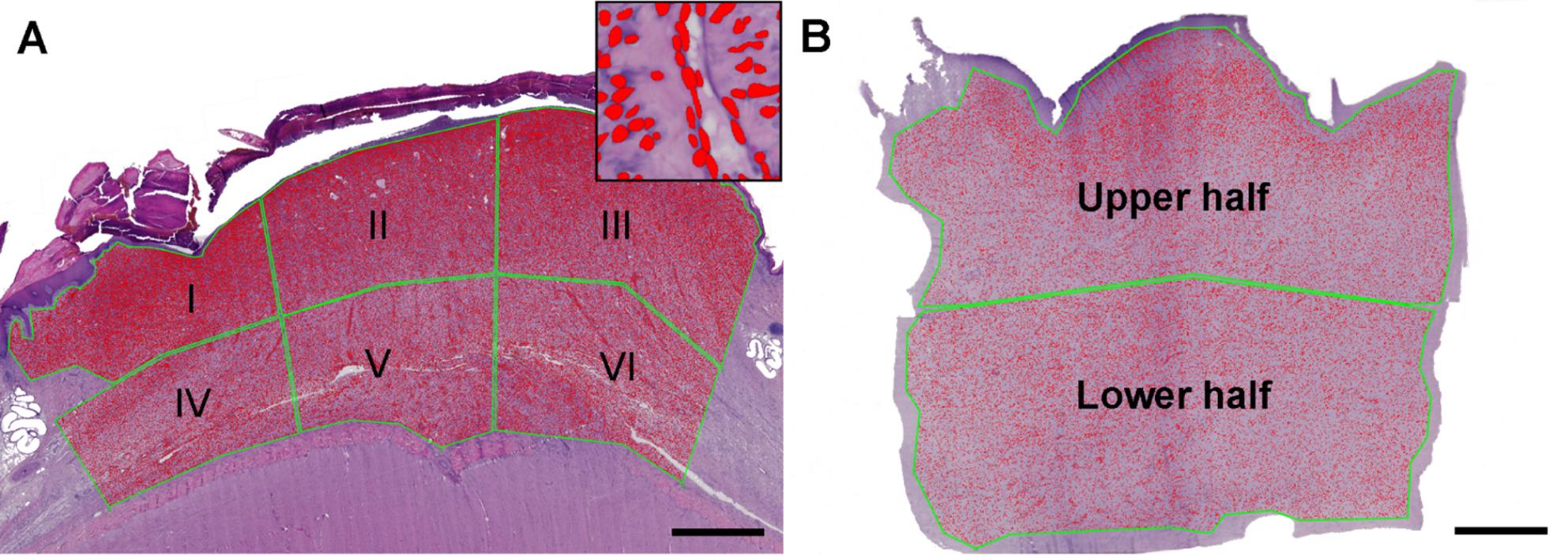
Detection and measurements of nuclei using the InstanSeg command in QuPath. **A**: Section of a 15-day-old experimental wound divided into six areas (I–VI). Bar = 4000 μm. Insert: High magnification showing the detected nuclei. **B**: Section of a wound from a forensic case, divided into an upper half and a lower half. The wound was located on an umbilical outpouching of a slaughter pig, and the estimated wound age was several weeks. Bar = 3000 μm

Each section of wound tissue sampled from the experimental wounds was divided into six areas (I–VI) as previously described [2, 9]. In brief, the granulation tissue was divided into an upper and lower half and further divided into six approximately equal-sized areas using the polygon annotation tool (Fig. 1A).

Each section of wound tissue from the forensic cases was divided into an upper and lower half. Forensic wounds could not be divided into six areas as wound borders were not necessarily present on the tissue slides. Measurement annotations were drawn at the center and periphery, and thereafter, a horizontal line was drawn dividing the wound tissue into two approximately equal halves. Then, the polygon annotation tool was used to define the two areas, i.e., the upper and lower half (Fig. 1B). The most superficial part of the wound, i.e., the wound surface, was not included in the upper area, as this tissue was affected by the slaughter procedure (Fig. 1B).

Nuclei were detected and measured using the InstanSeg command in QuPath with the settings shown in Table 1 (Fig. 1A and B). To validate this method, two experimental wounds were randomly selected from each age group using the RANDBETWEEN function in Excel. Within each selected wound, a 300 × 300 μm annotation was placed at the center of wound area II. Using the point annotation tool, all nuclei were manually counted and subsequently quantified by running the InstanSeg command. Thereafter, InstanSeg was used to quantify nuclei in all wound areas of the tissue sections. Measurements of nucleus area size (µm^2^), maximum diameter (µm), and circularity (0–1) were performed in experimental wound area II and in the upper half of the forensic wounds.

**Table 1:** InstanSeg settings for detection of nuclei in granulation tissue

Category	Option	Selected value
Model Selection	Model	Brightfield_nuclei
Annotations	Selection	All annotations selected
Additional Options	Preferred device	cpu
	Threads	4
	Tile size	512
	Tile padding	32
	Input channels	Red, green, and blue
	Outputs	All available
	Make measurements	Enabled
	Random colors	Disabled

The option “Make measurements” was enabled for the detection of nuclei in experimental wound area II and the superficial half of the granulation tissue from the forensic wounds only. For the remaining wound areas, this option was disabled

### Statistical analysis

Statistical analyses were conducted using a combination of Microsoft Excel (Microsoft 365, Microsoft Corporation, Washington, USA), GraphPad Prism (version 10.4.2., GraphPad Software, San Diego, CA, USA), and Python (version 3.12, The Python Software Foundation, www.python.org, Beaverton, Oregon, USA ).

Descriptive statistics were initially performed to summarize the datasets. For experimental wounds, the nuclei density values were pooled across areas I–III and IV–VI, respectively. Then nuclei density data (experimental and forensic wounds) were checked for normality using QQ plots and, if sample sizes permitted, the Shapiro-Wilk test, and were log-transformed if necessary. For group comparisons of nuclei density, a nested one-way analysis of variance (ANOVA) was conducted to evaluate differences among the eight experimental groups (seven age groups and one control group). Tukey’s multiple comparisons test was applied to identify specific differences between groups. Moreover, nested one-way ANOVA was used to identify differences between the three forensic age groups. Nested one-way ANOVA and Tukey’s multiple comparisons test were applied to identify differences in nuclei density between the three wound locations (umbilical outpouchings, limbs, and ear/tail).

Area, maximum diameter, and circularity datasets were checked for normal distribution using QQ plots and the Shapiro-Wilk test. The Kruskal-Wallis test was used to compare measurements of area size, maximum diameter, and circularity of the nuclei between the groups. More specifically, the tests were used to evaluate differences between the seven experimental age groups and differences between the three forensic age groups.

A significance level of α = 0.05, (*P* < 0.05) was used for all statistical tests, and effect sizes (eta-squared, η^2^_H_ and partial eta-squared, η^2^_P_) were calculated for each of the variables [16, 17].

## Results

### Wounds

In total, 181 out of 188 wounds were suitable for evaluation. Seven wounds were discarded due to artefacts or poor scanning quality, despite multiple attempts. In total, 1,068 wound areas (I-VI) were suitable for evaluation, while seventeen wound areas were not suitable for evaluation due to artefacts or poor scanning quality. All tissue sections of intact skin were evaluated (*n* = 13).

In total, 30 forensic wounds were included. Information regarding wound age, body location, thickness of granulation tissue, and fibrous tissue is presented in Table 2.

**Table 2:** Overview of forensic porcine wounds

Pig no. / characteristics	Wound age	Max length (cm)	Wound location	GT thickness (cm)	FST thickness (cm)	GT + FST thickness (cm)
1/ Slaughter pig*	Days	14	Head	0.2	0	0.2
1/ Slaughter pig	Days	7.0	Body	0	0	0
1/ Slaughter pig	Days	6.0	Limb	0	0	0
2/ Sow	Days	6.2	Unknown	0.1	0	0.1
2/ Sow	Days	4.6	Unknown	0.1	0	0.1
3/ Slaughter pig**	Days	5.0	Outpouching	0	0.5	0.5
			Average	**0.1**	**0.1**	**0.2**
4/ Slaughter pig	> 1 week	9.0	Limb	0.2	0.4	0.6
4/ Slaughter pig	> 1 week	2.0	Limb	0.4	1	1.4
5/ Slaughter pig*	> 1 week	7.5	Outpouching	0.4	0.3	0.7
6/ Slaughter pig	> 1 week	3.0	Tail	0.1	0.1	0.2
7/ Slaughter pig	> 1 week	3.2	Tail	NA	NA	0.7
8/ Slaughter pig	> 1 week	3.3	Tail	NA	NA	0.6
			Average	**0.3**	**0.5**	**0.7**
1/ Slaughter pig	Several weeks	5.0	Tail	NA	NA	0.3
9/ Slaughter pig**	Several weeks	15.0	Ear	1.1	1.5	2.6
10/ Slaughter pig	Several weeks	6.6	Limb	0.7	0.6	1.3
10/ Slaughter pig	Several weeks	1.9	Limb	NA	NA	0.7
10/ Slaughter pig	Several weeks	15.0	Limb	0.4	1.5	1.9
11/ Slaughter pig*	Several weeks	12.5	Outpouching	0.9	1	1.9
12/ Slaughter pig*	Several weeks	3.6	Tail	1	0	1
13/ Slaughter pig*	Several weeks	14.0	Outpouching	0.2	3.5	3.7
14/ Slaughter pig	Several weeks	5.0	Outpouching	0.5	3	3.5
14/ Slaughter pig**	Several weeks	3.5	Outpouching	0.4	1.5	1.9
15/ Slaughter pig	Several weeks	11.0	Outpouching	0.3	2.5	2.8
16/ Slaughter pig**	Several weeks	4.0	Outpouching	0.1	2	2.1
17/ Slaughter pig**	Several weeks	9.5	Outpouching	0.5	1.5	2
18/ Slaughter pig**	Several weeks	4.0	Outpouching	0.5	2.5	3
19/ Slaughter pig**	Several weeks	13.0	Limb	1	1.8	2.8
20/ Slaughter pig**	Several weeks	8.0	Tail	NA	NA	1
21/ Slaughter pig**	Several weeks	8.0	Outpouching	0.4	2	2.4
22/ Slaughter pig**	Several weeks	3.5	Outpouching	NA	NA	1
			Average	**0.6**	**1.8**	**2.0**

Wound age, location, and maximum (max) length, thickness of granulation tissue (GT), and fibrous scar tissue (FST) in porcine wounds received for forensic examination. For each age group (days, > 1 week, several weeks, and not stated), the average thicknesses of GT, FST, and GT + FST are presented. Wounds marked with* have previously been reported by Bækgård et al. [18], and wounds marked with ** have previously been reported by Pankoke et al. [2], but for other purposes

### Validation of InstanSeg

**Fig. 2:**
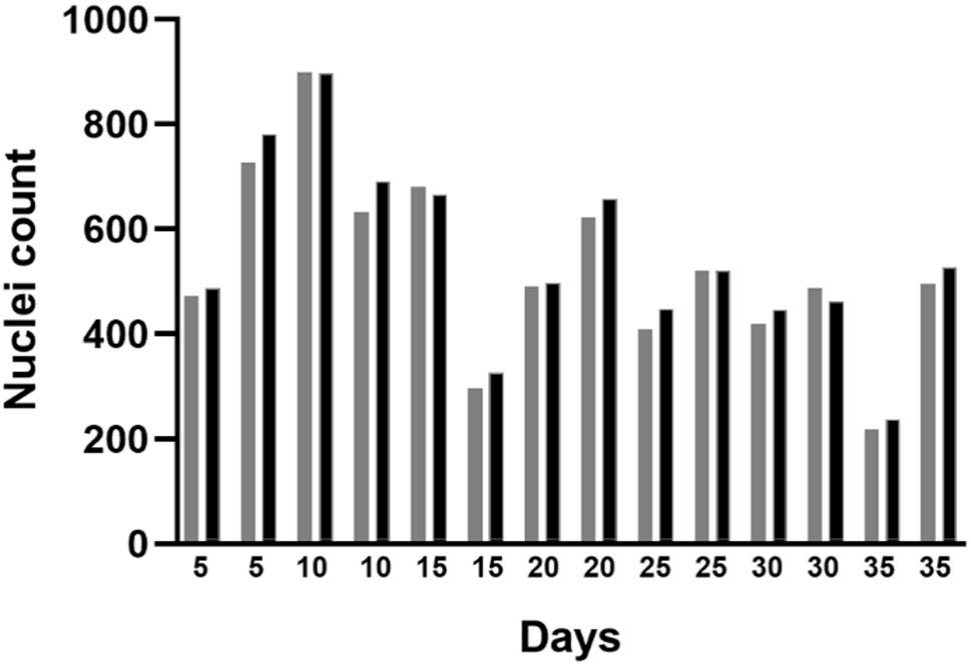
Validation of the nuclei detection performed by the InstanSeg command in QuPath. Numbers of nuclei detected manually (grey) and by InstanSeg (black) in a 300 × 300 μm annotation (ROI) placed at the center of wound area II. Two wounds were selected from each age group. Using the point annotation tool, all nuclei were manually counted in the ROI and subsequently quantified by running the InstanSeg command

In the validation areas, the manual counting identified a total of 7,372 nuclei, while the InstanSeg algorithm detected 7,640 nuclei, i.e., a difference of 3.6%. The numbers of nuclei in the individual validation areas are presented in Fig. 2. In 12 out of the 14 validation areas (86%), InstanSeg identified more, or equal numbers of nuclei compared to the manual counting. The difference in counts was not related to a specific cell type.

### Nuclei density

**Fig. 3:**
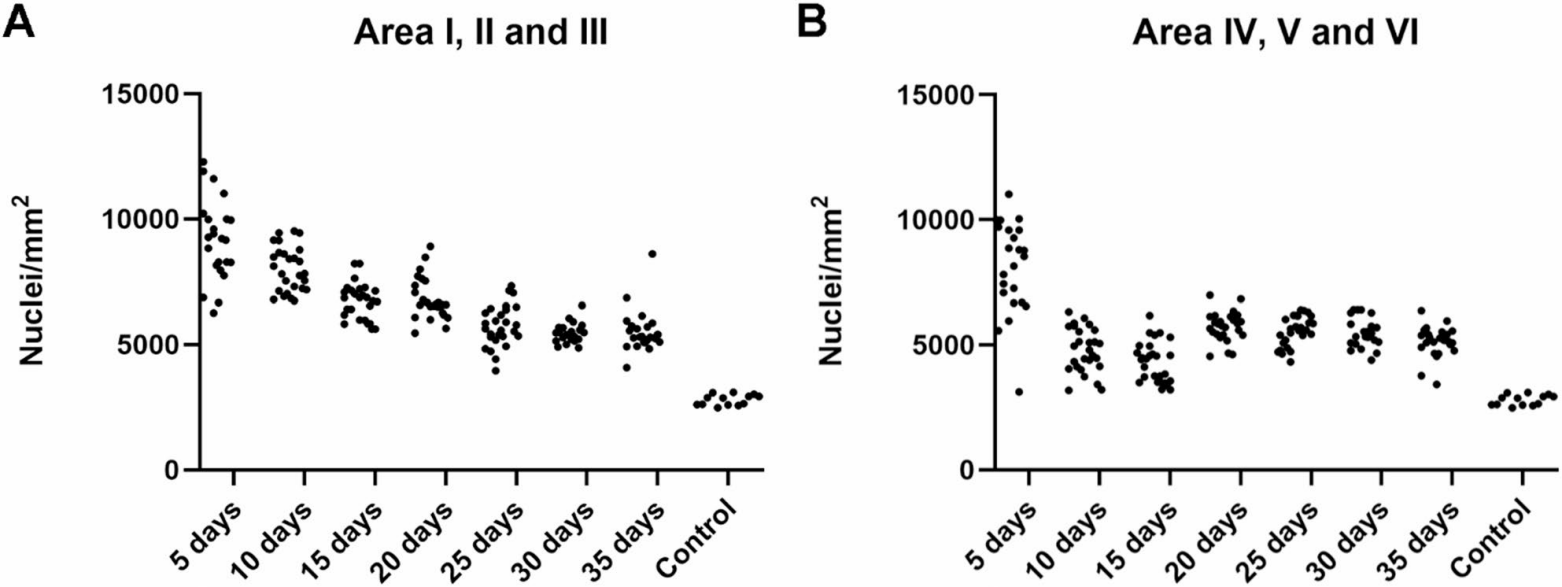
Nuclei density in experimental wounds. Nuclei per mm^2^ in experimental wounds ranging from 5 days up to 35 days old and intact skin (control). **A**: Nuclei per mm^2^ in the upper part of the experimental wound (wound areas I–III). **B**: Nuclei per mm^2^ in the deep part of the experimental wound (wound areas IV–VI)

The density of nuclei in the experimental wounds showed a similar time pattern in wound areas I–III and IV–VI, respectively (Additional file 1). Therefore, the density values for wound areas I–III and IV–VI, respectively, were pooled (Fig. 3A and B).

The log-transformed data from the experimental wounds were normally distributed. The density of nuclei decreased with increasing wound age, and statistically significant differences were present between the groups both in the upper half (*P* < 0.05, η^2^_P_ = 0.94) and lower half (*P* < 0.05, η^2^_P_ = 0.88) of the wounds. Moreover, significant differences were found between many of the age groups in both wound areas I–III and IV–VI, respectively (Table 3). However, these time-dependent differences were most apparent in the superficial part of the wounds (wound areas I–III) (Fig. 3A).

**Table 3:** Comparisons of nuclei density between experimental wounds (5–35 days old) and intact skin

Comparison	Wound area I–III	Wound area IV–VI
	*P*-value	*P*-value
5 days vs. 10 days	0.06	< 0.05*
5 days vs. 15 days	< 0.05*	< 0.05*
5 days vs. 20 days	< 0.05*	< 0.05*
5 days vs. 25 days	< 0.05*	< 0.05*
5 days vs. 30 days	< 0.05*	< 0.05*
5 days vs. 35 days	< 0.05*	< 0.05*
5 days vs. Control	< 0.05*	< 0.05*
10 days vs. 15 days	< 0.05*	0.32
10 days vs. 20 days	< 0.05*	< 0.05*
10 days vs. 25 days	< 0.05*	< 0.05*
10 days vs. 30 days	< 0.05*	0.05
10 days vs. 35 days	< 0.05*	0.64
10 days vs. Control	< 0.05*	< 0.05*
15 days vs. 20 days	> 0.99	< 0.05*
15 days vs. 25 days	< 0.05*	< 0.05*
15 days vs. 30 days	< 0.05*	< 0.05*
15 days vs. 35 days	< 0.05*	< 0.05*
15 days vs. Control	< 0.05*	< 0.05*
20 days vs. 25 days	< 0.05*	> 0.99
20 days vs. 30 days	< 0.05*	0.98
20 days vs. 35 days	< 0.05*	0.24
20 days vs. Control	< 0.05*	< 0.05*
25 days vs. 30 days	0.95	> 0.99
25 days vs. 35 days	0.97	0.65
25 days vs. Control	< 0.05*	< 0.05*
30 days vs. 35 days	> 0.99	0.84
30 days vs. Control	< 0.05*	< 0.05*
35 days vs. Control	< 0.05*	< 0.05*

For group comparisons of nuclei density Tukey’s multiple comparisons test was applied to identify specific differences between experimental wounds (5–35 days old) and intact skin. P-value for each comparison is listed. Significant differences (*P* < 0.05)* were found between many of the age groups in both wound areas I–III and IV–VI

**Fig. 4:**
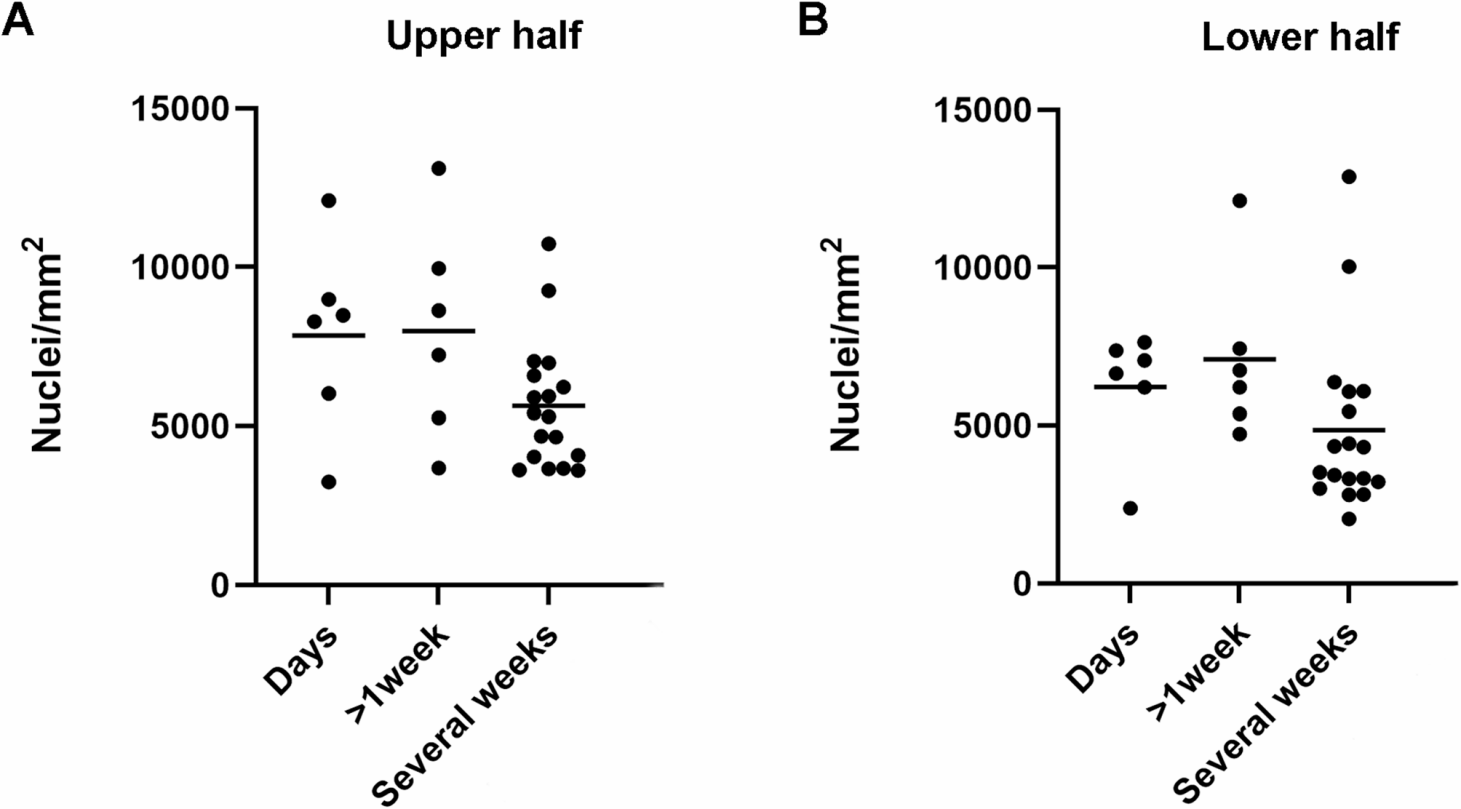
Nuclei density in forensic wounds. Nuclei per mm^2^ in forensic wounds in each of the three age groups (days, > 1 week, several weeks). The mean is shown as a horizontal line. **A**: Nuclei per mm^2^ in the upper half of the wound. **B**: Nuclei per mm^2^ in the lower half of the wound

Nuclei density data from the forensic wounds were normally distributed. No statistically significant differences in nuclei density were found between the age groups in either the upper half (*P* = 0.12, η^2^_P_ = 0.19) or the lower half (*P* = 0.27, η^2^_P_ = 0.12) of the granulation tissue (Fig. 4A and B).

**Fig. 5:**
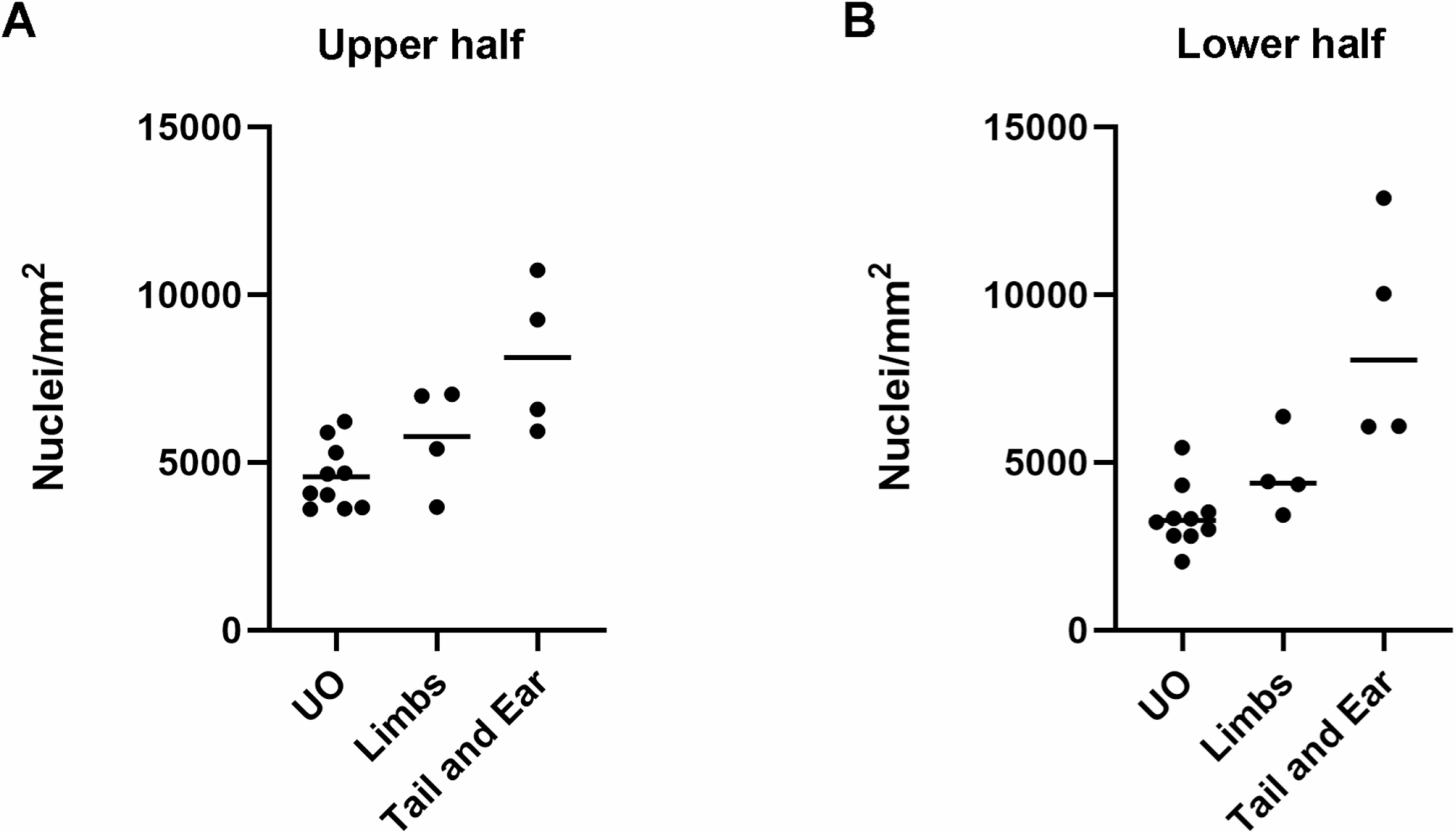
Nuclei density in wounds located on umbilical outpouchings (UO), limbs, and ear/tail. Nuclei per mm^2^ in forensic wounds, several weeks old, in each of the three wound locations, i.e., UO, limbs, and ear/tail. The mean is shown as a horizontal line. **A**: Nuclei per mm^2^ in the upper half of the wound. **B**: Nuclei per mm^2^ in the lower half of the wound

A significant difference was found between wounds located on different body parts in both the upper half (*P* < 0.05, η^2^_P_ = 0.59) and the lower half (*P* < 0.05, η^2^_P_ = 0.69) of the wound tissue (Fig. 5A and B). More specifically, wounds located on umbilical outpouchings differed from wounds on ears/tails in both the upper (*P* < 0.05) and lower (*P* < 0.05) halves of the wound tissue. Moreover, a significant difference was found between forensic wounds located on limbs and ears/tails in the lower half of the wound tissue (*P* < 0.05).

### Nuclear dimensions and circularity

Descriptive statistics for each of the experimental age groups and forensic age groups are presented in Tables 4 and 5, respectively. Significant differences (*P* < 0.05) were found between the experimental groups (5–35 days) for all three variables, i.e., area, diameter, and circularity of nuclei. However, effect sizes were η^2^_H_ = 0.01, η^2^_H_ = 0.02, and η^2^_H_ = 0.01 for the area, diameter, and circularity of nuclei, respectively. Moreover, significant differences (*P* < 0.05) were found between the forensic age groups for all three variables, i.e., area, diameter, and circularity of nuclei. However, effect sizes were η^2^_H_ = 0.03, η^2^_H_ = 0.03, and η^2^_H_ = 0.003 for the area, diameter, and circularity of nuclei, respectively.

**Table 4:** Measurements of nucleus dimensions (area, diameter, and circularity) in granulation tissue from experimental wounds

Wound age	Area, µm^2^			Diameter, µm			Circularity		
	No. of nuclei	Median (IQR)	Mean	No. of nuclei	Median (IQR)	Mean	No. of nuclei	Median (IQR)	Mean
5 days	1,841,138	23.99 (16.00-36.75)	28.26	1,841,138	7.44(5.84–9.63)	8.00	1,838,445	0.55 (0.49–0.60)	0.55
10 days	4,872,902	25.74 (17.10-38.88)	29.90	4,872,903	8.13 (6.35–10.40)	8.67	4,870,280	0.54 (0.46–0.59)	0.52
15 days	4,159,588	27.25 (18.25–40.51)	31.34	4,159,589	8.38 (6.57–10.74)	8.96	4,157,154	0.53 (0.46–0.59)	0.52
20 days	2,623,307	27.00 (18.00-39.74)	30.58	2,623,308	8.38 (6.55–10.74)	8.97	2,621,461	0.53 (0.45–0.59)	0.51
25 days	2,157,680	28.25 (18.5–41.50)	31.99	2,157,680	8.75 (6.80-11.24)	9.34	2,156,154	0.52 (0.43–0.58)	0.50
30 days	1,354,496	28.01 (18.25-41.00)	31.56	1,354,498	8.90 (6.82–11.42)	9.47	1,353,422	0.51 (0.42–0.58)	0.49
35 days	1,182,238	28.50 (18.99–41.33)	31.98	1,182,238	8.86 (6.95–11.40)	9.49	1,181,027	0.51(0.42–0.58)	0.49

Number of nuclei, median, interquartile range (IQR), and mean are shown for each of the seven age groups (5–35 days)

**Table 5:** Nucleus dimensions in granulation tissue from porcine wounds received for forensic examination

Wound age	Area, µm^2^				Diameter, µm				Circularity, 0–1			
	No. of nuclei	Median (IQR)	Mean	No. of nuclei		Median (IQR)	Mean	No. of nuclei		Median (IQR)	Mean	
Days	976,350	21.50 (15.00-30.75)	24.45	976,351		8.06 (6.32–10.29)	8.57	975,607		0.49 (0.40–0.56)	0.48	
> 1 week	1,633,853	16.75 (12.01-23.00)	18.89	1,633,853		6.73 (5.43–8.38)	7.23	1,632,923		0.51 (0.43–0.58)	0.50	
Several weeks	10,743,687	22.49 (15.24-33.00)	25.99	9,724,167		8.06 (6.31–10.20)	8.55	10,740,513		0.50 (0.41–0.57)	0.49	

Measurements of nucleus dimensions (area, diameter, and circularity) in granulation tissue from porcine wounds received for forensic examination. Number of nuclei, median, interquartile range (IQR), and mean shown for each of the three age groups (days, > 1 week, and several weeks)

## Discussion

The density of nuclei in granulation tissue from experimental wounds displayed a time-dependent pattern, where the density decreases as the wound age increases. This pattern is in accordance with previous studies of wound healing in which cell proliferation and infiltration of leukocytes are present during early wound healing and decrease in the maturation phase [9, 12]. In forensic cases, lesion age is typically stated in vague estimates such as days, weeks, or months [1], therefore, all improvements in accuracy are warrant for the assessment of age. A time-dependent pattern in nuclei density was especially apparent in the upper half of the experimental wound. This finding indicates that nuclei density, at least in the upper half of the wound, may provide an objective tool for age assessment in a forensic setting. However, due to overlap in nuclei density between age groups, this measure should be used in combination with other methods, such as gross and histological evaluation of wounds [8]. Immunohistochemistry and flow cytometry have been used to quantify cells expressing CD34, CD45, and CD105 in experimental wounds [18]. The percentage of CD45- and CD105-positive cells showed a time-dependent pattern; however, these markers alone were not sufficient as parameters for determining wound age [18]. Moreover, these methods may be challenging to apply in a forensic context. Flow cytometry requires viable cells, which means that sampling must be performed shortly after the death of the animal, and immunohistochemical labelling likewise often depends on tissue of high quality [2, 18]. HE staining of sections is routinely carried out in many forensic post-mortem examinations. Therefore, nuclei density measurements can be performed with no additional cost when using open-source software.

Generally, InstanSeg quantified more or equal numbers of nuclei compared to manual counting. Moreover, InstanSeg was found suitable for nuclei quantification in porcine granulation tissue, as manual and InstanSeg counts were fairly similar (Fig. 2). The morphological appearance of granulation tissue in wounds healing by second intention varies across different regions of the wound [18]. Typically, the granulation tissue nearest to the wound surface appears more immature compared to the more established granulation tissue in the deepest part [18]. To account for this, we divided wounds into upper and lower halves. However, this division may be somewhat imprecise and affected by the angle of sectioning. Despite this limitation, the nuclei density in the upper wound areas (I–III) and lower wound areas (IV–VI), respectively, showed the same time-dependent pattern (Additional file 1).

In granulation tissue of forensic wounds, no significant difference in nuclei density was found between the three age groups. Even though non-significant, there seems to be a tendency towards a time-dependent pattern, with the nuclei density decreasing as wound age estimates increase (Fig. 4A and B). This variation within the groups may be due to other factors such as wound location, size, the age of the pig, the thickness of the granulation tissue, handling of the tissue prior to sampling, and a limited sample size. In the forensic cases, the majority of pigs were slaughter pigs, approximately 5–6 months old. However, the thickness of the granulation tissue in the forensic wounds did show variation within the groups, e.g., in the age group “several weeks” the sum of granulation tissue and underlying fibrous tissue varies from 0.3 to 3.7 cm, underlining the high variability between the forensic wounds. The majority of the forensic cases were processed at the slaughterhouse, and all cases were frozen prior to sampling. The slaughter procedure removes the most superficial part of the skin and wound tissue, which might also have influenced the results. Moreover, freezing and thawing tissue before histological evaluation introduces artefacts that may have destroyed an unknown number of cells.

The location of the wound on the body might also influence the nuclei density as factors such as blood supply, mechanical stress, and infection are known to affect the wound healing process [19, 20]. In the present study, 12 out of 30 (37.5%) forensic wounds were located on umbilical outpouchings, i.e., umbilical hernia and enterocystoma. From 2004 to 2016, 59 out of 217 (27.2%) wounds submitted for forensic examination at the University of Copenhagen were located on umbilical outpouchings [4]. Based on this number, there seems to be an overrepresentation of porcine wounds located on umbilical outpouchings, which are relatively common in Danish weaners with an average herd prevalence of 2.9% [21]. Nuclei density was significantly lower in forensic wounds located on umbilical outpouchings compared to wounds on the ears/tails. In humans, wound location has been shown to influence the healing rates of wounds on the lower extremities [22]. Moreover, in horses, limb wounds have been found to heal at a reduced rate and with increased risk of complications compared to wounds located on the body [23]. In an experimental equine wound model, tissue oxygen saturation was shown to be lower in the limb wounds compared to wounds on the body, indicating that blood supply affects the healing process [19]. Therefore, it could be speculated that wounds on the skin covering umbilical outpouchings may show delayed wound healing and, therefore, appear more often in forensic cases compared to wounds on other body locations. The nuclei density of the forensic wounds was affected by the location of the wound, perhaps due to differences in blood supply or other healing conditions in certain locations on the body. However, a parameter such as oxygen saturation has not been investigated in porcine skin covering umbilical outpouchings. Overall, the results show that nuclei density in wounds is affected by other factors, such as the location on the body, that can mask the age-related pattern. Therefore, measurements of nuclei density should be used with caution and always in combination with other methods when assessing wound age.

In future studies, the experimental model should be fully validated using wounds of known age, located at various locations on the body. Preferably, such wounds should be non-experimental and sampled shortly after death of the animal. However, given the circumstances of forensic cases, the exact age of a wound is usually not available.

Measurements of nucleus area, diameter, and circularity showed a subtle time-dependent pattern (Tables 4 and 5). Even though the differences were found to be statistically significant, the effect sizes were between 0.003 and 0.03. In very large datasets, non-parametric tests such as the Kruskal-Wallis can detect small differences between groups which may not be of biological relevance [24]. Indeed, the small effect sizes suggest that wound age accounts for only a minor proportion of variation in area size, diameter, and circularity of nuclei.

## Conclusion

The density of nuclei in experimental wounds displayed a significant time-dependent pattern, where the density decreased as the wound age increased. In forensic wounds, nuclei density showed a tendency of a time-dependent pattern, but it was not significant. Based on this, digital quantification of nuclei is promising as a supportive tool for objective age assessment of wounds, but should be used in combination with other methods, such as gross and histological evaluation. Nuclei density measurements cannot yet be used as a standalone method as the nuclei density may also be affected by the location of the wound. Measurements of nucleus area, diameter, and circularity were not found to be suitable for age assessment of wounds.

## Supplementary Information

Below is the link to the electronic supplementary material.

Supplementary Material 1.

## Data Availability

The data analyzed are available from the corresponding author on reasonable request.
