# Supplementary figures and images for: Digital quantification of nuclei in porcine experimental and forensic wounds: effects of wound age and body location on nuclei density
Source: Acta Vet Scand. 2026 Apr 29;68:28. doi: 10.1186/s13028-026-00866-5 (PMC13330206; doi:10.1186/s13028-026-00866-5)

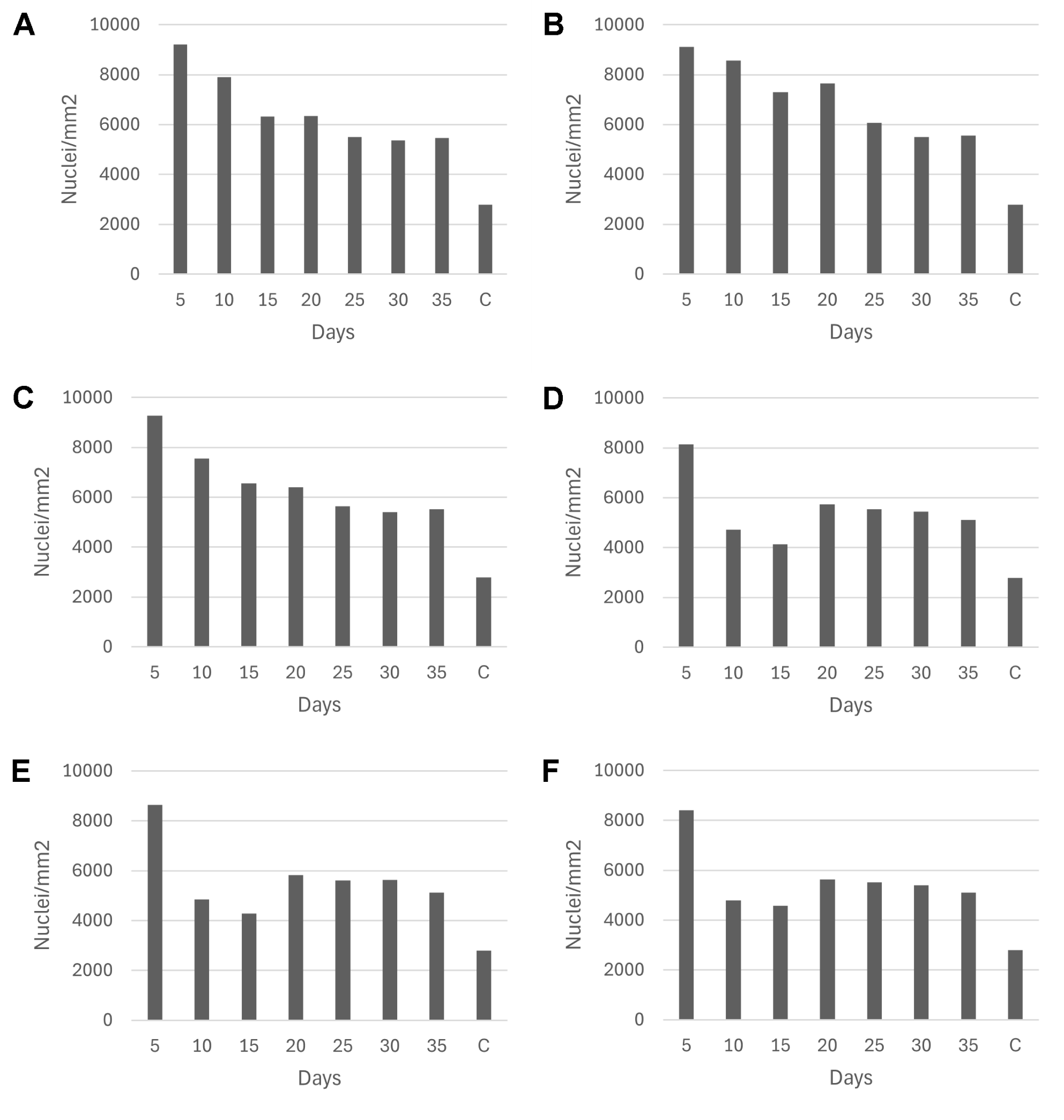

Supplement: Supplementary file 1 — Supplementary Material 1. [file 13028_2026_866_MOESM1_ESM.tif]
